# circFADS2 protects LPS-treated chondrocytes from apoptosis acting as an interceptor of miR-498/mTOR cross-talking

**DOI:** 10.18632/aging.101986

**Published:** 2019-05-29

**Authors:** Guoqing Li, Wei Tan, Yuxuan Fang, Xia Wu, Wei Zhou, Chunwang Zhang, Yu Zhang, Yanqing Liu, Guangzheng Jiu, Dan Liu

**Affiliations:** 1 Department of Rheumatology, Affiliated Hospital of Yangzhou University, Yangzhou University, Jiangsu 225000, China; 2 Clinical Medical College, Dalian Medical University, Jiangsu 225000, China; 3 Medical College of Yangzhou University, Jiangsu 225000, China; 4 Department of Physiology, School of Medicine, Showa University, Jiangsu 225000, China; 5 Department of Pathology, Clinical Medical College, Yangzhou University, Jiangsu 225000, China

**Keywords:** rheumatoid arthritis, circFADS2, miR-498, mTOR, apoptosis

## Abstract

We aimed to investigate the regulation of circular RNAs in lipopolysaccharide (LPS)-treated chondrocytes isolated from SD rat. In this study, we analyzed how circFADS2 was regulated in LPS-treated chondrocytes and isolates from Rheumatoid arthritis (RA) patients and found that circFADS2 and mTOR were highly expressed whereas miR-498 expression was significantly reduced. We then silenced circFADS2 in LPS-treated chondrocytes; this resulted in a declined expression of type II collagen, but an increase in the expression of MMP-13, COX-2, and IL-6. Overall, silencing circFADS2 caused a significant reduction in the proliferative rate of LPS-treated chondrocytes, increased apoptotic levels, miR-498 upregulation, and mTOR downregulation. Dual-luciferase reporter assay indicated that circFADS2 directly targeted miR-498. In contrast, miR-498 down-regulation affected circFADS2 silencing, promoting extracellular matrix (ECM) degradation and apoptosis. The 3’ UTR of the mTOR gene is targeted by miR-498, and consequently, in cells transfected with miR-498, there was a significant reduction of mTOR expression at the protein and mRNA levels. Silencing mTOR had a similar effect to circFADS2 silencing on type II collagen, MMP-13, COX-2, and IL-6 expression, as well as cell proliferation and apoptosis. In conclusion, circFADS2 may affect LPS-induced chondrocytes properties by regulating the ECM catabolism, inflammation, and apoptosis in chondrocytes.

## INTRODUCTION

Rheumatoid arthritis (RA) is a chronic inflammatory disorder whose major features are hypertrophy, hyperplasia, and angiogenesis in synovial tissues [1, 2]. RA pathogenesis involves intrinsic and extrinsic factors [3]. It is estimated that about 1% of the global population suffers from this condition that causes a dramatic decline in life quality [4]. Emerging evidence has demonstrated that cytokine networks and associated cells are two major factors involved in RA pathogenesis. Extracellular matrix (ECM) proteolysis in articular cartilage is crucial to RA development [5]. Generally, ECM degradation is caused by a massive release of inflammatory cytokines, including prostaglandins, ROS, and proinflammatory cytokines [6]. Nevertheless, the details involving RA pathogenesis remain poorly understood.

Circular RNAs (circRNAs) are RNAs with loop structures that are generated by aberrant splicing [7, 8]. Increasing evidence has shown that circRNAs are critical to some biological events, such as cell proliferation and migration among others [9, 10]. It has also been documented that circRNAs play a role in different types of arthritis, including osteoarthritis (OA) and RA. For instance, Wu et al. indicated that in the presence of miR-26a, circRNA hsa_circ_0005105 up-regulates NAMPT causing an increase in ECM proteolysis in OA chondrocytes [11]. Ouyang et al. determined the circRNAs present in peripheral blood mononuclear cells (PBMCs) in RA patients and found five up-regulated circRNAs that could serve as potential biomarkers in RA diagnosis [12]. Nevertheless, circRNA expression and regulatory mechanism in RA chondrocytes are rarely reported.

MicroRNAs (miRNAs) can alter mRNA expression and consequently, the expression of specific genes [13, 14]. Recent studies showed that circRNA could bind to miRNAs and repressing their function, and modulate gene transcription in arthritis. Li et al. showed that Hsa_circ_0045714 is implicated in abnormal biological events that occur in OA chondrocytes by acting on the miR-193b [15]. Circular RNA Atp9b has been proved to influence OA acceleration by competing with miR-135-5p [16]. Additionally, miRNAs also play a direct and essential role in RA. Chatzikyriakidou et al. reviewed the differently expressed miRNAs in various RA-affected tissues [17] and found a relevant polymorphism that is probably associated with pathogenesis of RA or other autoimmune diseases.

It has been reported that the up-regulation of circFADS2 is associated with various pathological conditions, including advanced TNM stage. Previous mechanical studies showed that circFADS2 inhibition suppressed lung cancer cell proliferation, migration, and invasion abilities and that circFADS2 acted as a miR-498 sponge mediating tumorigenicity [18]. The present experimental data suggested that circFADS2 upregulation in chondrocytes of RA patients and LPS-treated chondrocytes can promote mTOR expression and protect cells from apoptosis by acting as a miR-498 sponge.

## RESULTS

### CircRNA expression profile in the cartilage tissues from RA patient and healthy control

**Figure 1:**
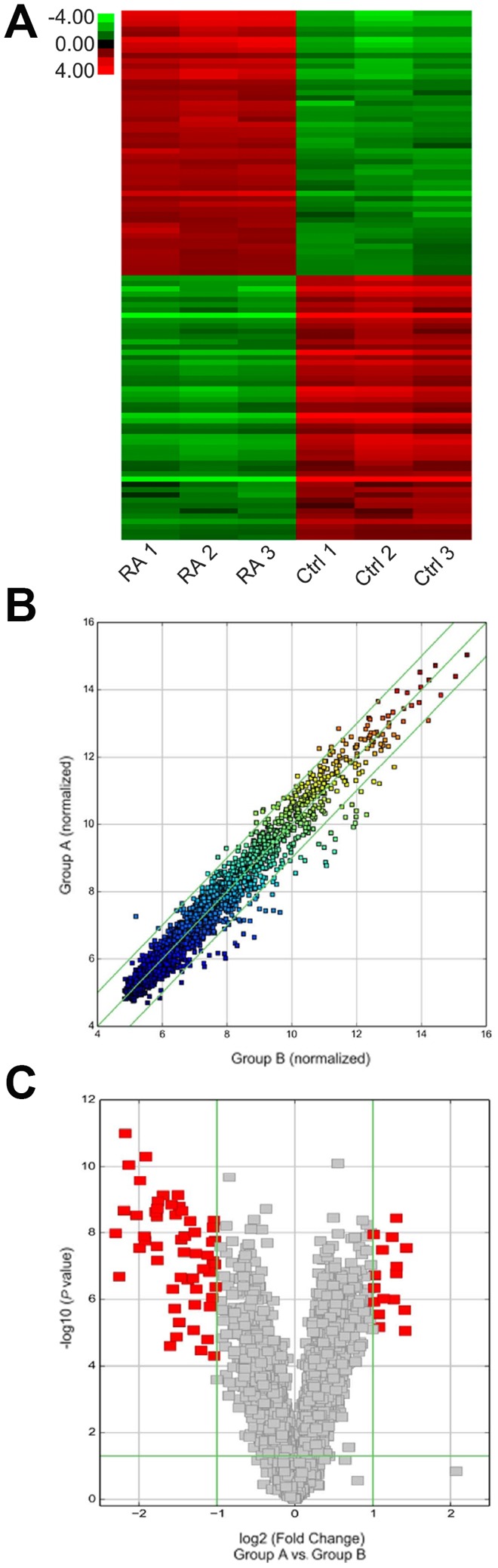
**Expression of various circRNAs in the cartilage tissue of RA patients.** (**A**) Hierarchical clustering assessment of circRNAs that displayed variation in expression patterns between control and RA groups; every group comprised three individuals (over two-fold difference in expression; P < 0.05). Expression values are shown in various colors suggestive of high and low median expression levels. (**B**) Scatter plot was used to evaluate alterations in circRNA expression between control (group A) and RA (group B) specimens. Values corresponding to X and Y axes in the scatter plot were normalized signal values of specimens (log_2_ scaled). Green lines indicate fold alterations. CircRNAs over the top green line and below the bottom green line indicate over two-fold changes. (**C**) Volcano plots were built to show fold change values and P values. The vertical lines show two-fold upregulation and downregulation between control and RA specimens (A versus B), and the horizontal line shows P value. The red point in the plot shows various expression patterns of circRNAs with statistical significance.

The expression of circRNA was evaluated in RA cartilage tissue and control specimens with hierarchical clustering (Figure 1A). Change in circRNA expression between RA cartilage tissue and control specimens was revealed via scatter and volcano plots (Figure 1B, 1C). Thirty-six circRNAs showed remarkable expression patterns in RA specimens as compared with the control specimens. Of these, 21 circRNAs were downregulated and 15 candidates showed upregulated expression in RA specimens. CircFADS2 showed the most obvious variation in expression.

### Effects of circFADS2 on LPS-treated chondrocytes

**Figure 2:**
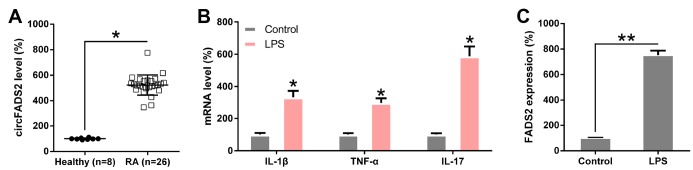
**circFADS2 is upregulated in RA patients and LPS-treated chondrocytes.** (**A**) qPCR analysis showing upregulated circFADS2 expression levels in RA patients. (**B**) qPCR analysis showed expression of IL-1β, TNF-α, and IL-17 in LPS-treated chondrocytes. (**C**) CircFADS2 levels were significantly augmented in LPS-treated chondrocytes when compared to the control group (non-treated cells). *P < 0.05, **P < 0.01 vs. the indicated group.

To confirm and uncover the role of circFADS2 in chondrocytes after LPS treatment, we first confirmed the difference in circFADS2 expression in RA patients and LPS-treated chondrocytes vs. the control groups by qPCR analysis. CircFADS2 expression was significantly upregulated in the samples of the 26 RA patients when compared to the healthy control (Figure 2A). To confirm the effect of LPS treatment on RA cell model, we firstly examined the expression of these three cytokines (IL-1β, TNF-α, and IL-17), qPCR data indicated that IL-1β, TNF-a, and IL-17 were upregulated (Figure 2B), indicating that these cytokines, especially IL-1β and TNF-α (two key RA model inducers), may also be participated in this regulation. LPS-treated chondrocytes exhibited increased circFADS2 levels when compared to untreated chondrocytes (Figure 2C), revealing that circFADS2 plays a critical role in LPS-response in the chondrocytes.

**Figure 3:**
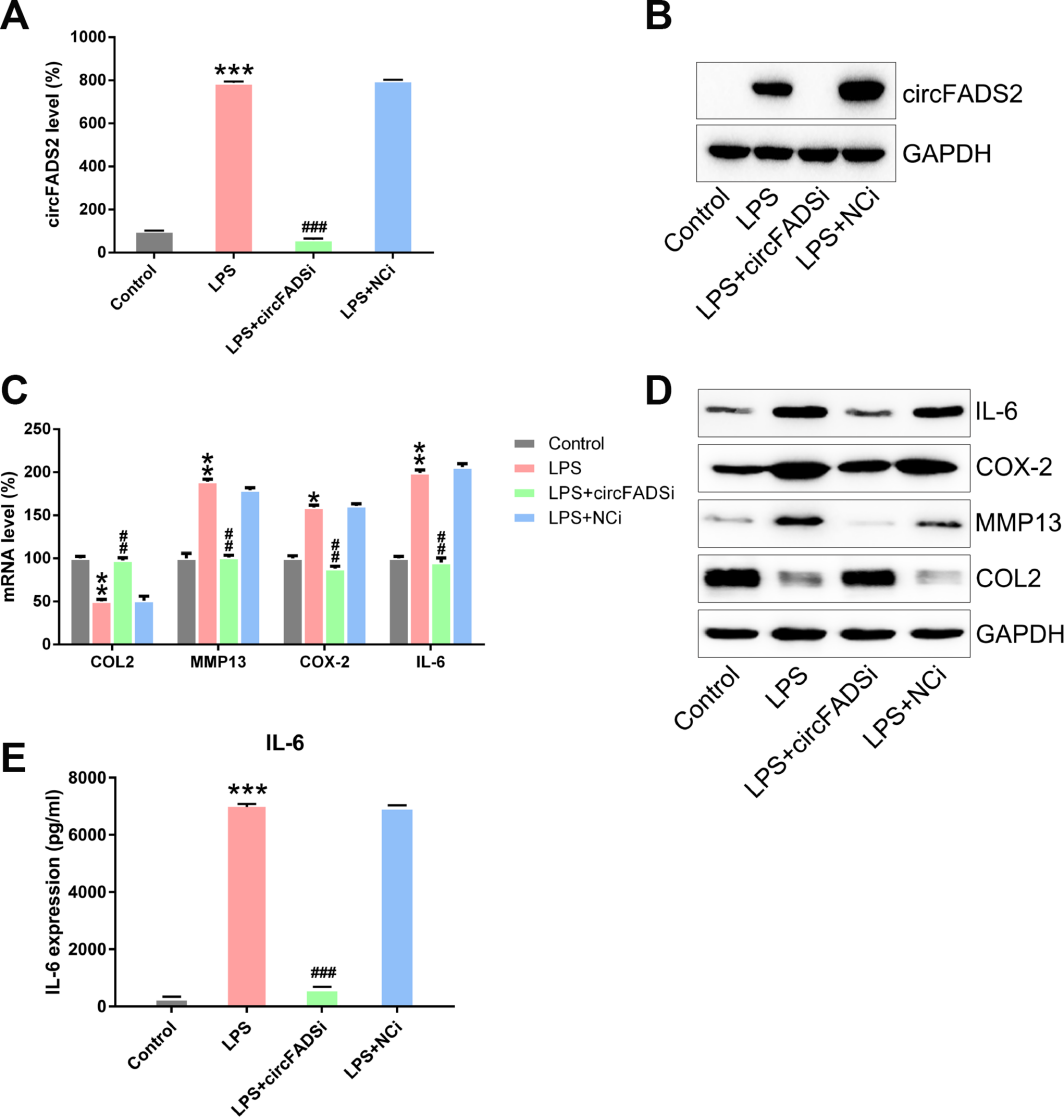
**circFADS2 regulates ECM degradation and inflammation of LPS-treated chondrocytes.** Chondrocytes were transfected with si-circFADS2 or si-NC, followed by LPS-treatment. (**A**) qPCR analysis was carried out to detect the levels of circFADS2 in each group. (**B**) Quantification of circFADS2 and GAPDH mRNA by northern blot analysis. (**C**) mRNA expression of the indicated proteins measured by qPCR; the results are normalized to the expression of GAPDH. (**D**) Western blotting revealing the expression of the indicated proteins; results are normalized to the expression of GAPDH. The protein levels of type II collagen, MMP13, COX-2, and IL-6 are shown in the WB-graph. (**E**) The IL-6 level in cells was examined by ELISA. *P < 0.05, **P < 0.01, ***P < 0.001 vs. control group, ^##^ P < 0.01, ^###^ P < 0.001 vs. LPS group.

To reveal how circFADS2 affects ECM catabolism and inflammation in chondrocytes after LPS treatment, we transfected the cells with a siRNAs against circFADS2 (si-circFADS2) to suppress circFADS2 expression and explored the downstream effects of this down-regulation on LPS-treated chondrocytes. qPCR and northern blot results confirmed that circFADS2 was remarkably down-regulated after si-circFADS2 transfection when compared to the NC group (Figure 3A, 3B). ELISA, qPCR and WB analyses showed that circFADS2 knockdown produced higher levels of type II collagen (COL2) when compared to the WT group and down-regulated MMP13, COX-2, and IL-6 at the RNA and protein level in LPS-treated chondrocytes (Figure 3C–3E). Thus, circFADS2 knockdown protects the chondrocytes against ECM degradation and massive release of inflammatory cytokines after LPS-treatment.

**Figure 4:**
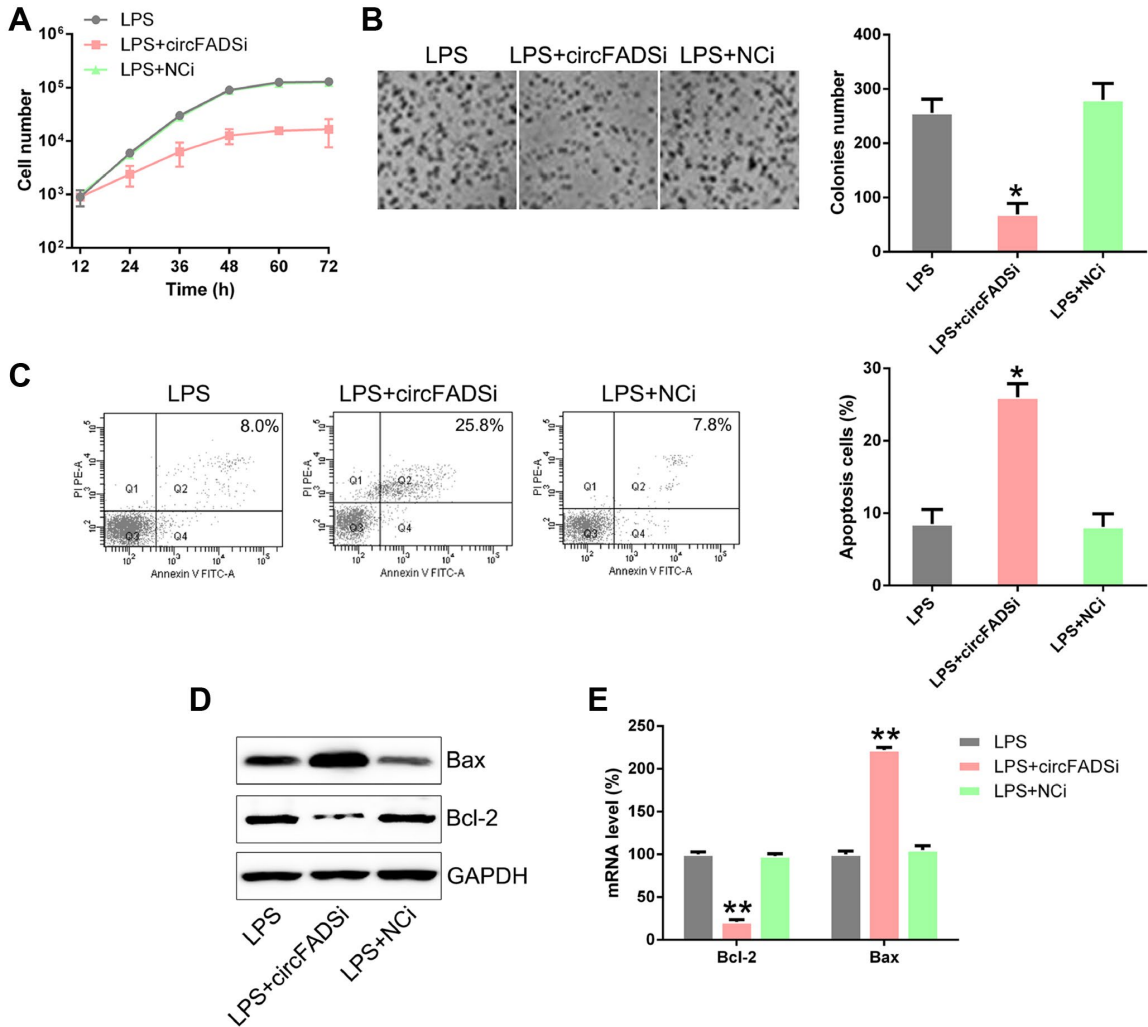
**circFADS2 silencing reduced cell viability and induced apoptosis in LPS-treated chondrocytes.** (**A**) CCK8 assay showing that transfection with a circFADS2 silencer (si-circFADS2) inhibited cell proliferation of LPS-treated chondrocytes. (**B**) Soft agar colony formation assay for the LPS-treated chondrocytes transfected with si-circFADS2 or si-NC, and that of non-transfected cells. The right panel shows the number of colonies formed in each group. (**C**) Flow cytometry analysis showing the levels of apoptosis in the different groups of chondrocytes. (**D**) WB and (**E**) qPCR analysis showing that circFADS2 inhibition downregulates Bcl-2 and up-regulates Bax in LPS-treated chondrocytes. *P < 0.05, **P < 0.01 vs. LPS group.

To explore whether circFADS2 regulated the proliferation and apoptosis of the chondrocytes after LPS treatment, we inhibited circFADS2 expression (Figure 3A) and measured the changes in cell viability and proliferation by the CCK-8 and colony formation assays. The results showed that after circFADS2 suppression, cell viability and proliferation were reduced when compared to the WT and NC groups (Figure 4A, 4B). We measured the apoptotic levels of LPS-induced chondrocytes with or without circFADS2 silencing and found that circFADS2 depletion resulted in increased apoptotic levels (Figure 4C). When compared to the WT group, the circFADS2-silenced group showed a decrease in Bcl-2, but an increase in Bax at the protein and mRNA levels (Figure 4D, 4E). In conclusion, circFADS2 protects chondrocytes against apoptosis and production of apoptotic factors.

### CircFADS2 directly targets miR-498

**Figure 5:**
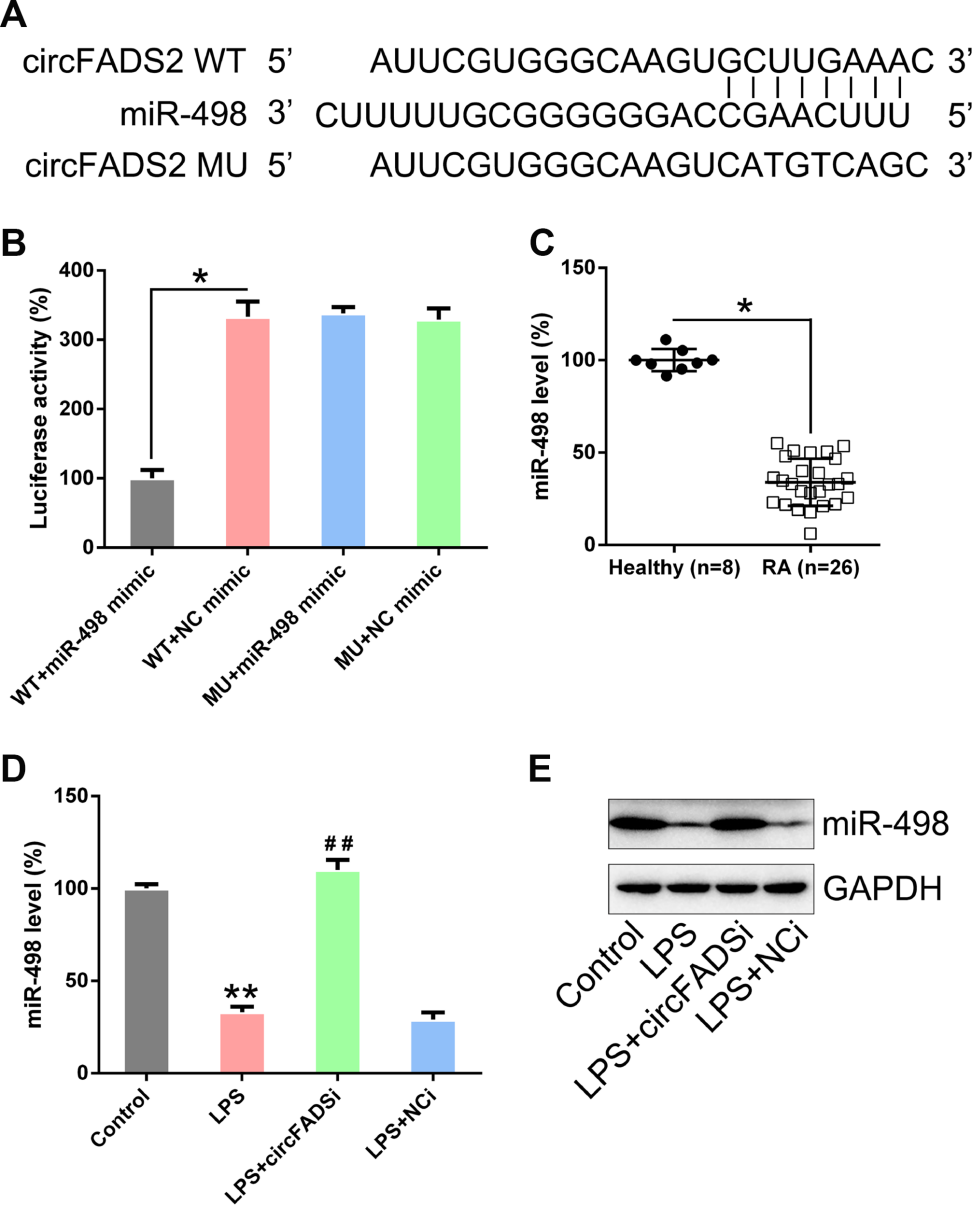
**circFADS2 targets miR-498.** (**A**) Bioinformatic analysis indicating that miR-498 has a circFADS2 binding site. (**B**) DLRA was performed following co-transfection of chondrocytes with a luciferase reporter containing either a WT (wild-type) or MU (mutant) circFADS2 and a miR-498 mimic. (**C**) qPCR analysis showing the decreased miR-498 expression levels in RA patients. (**D**) Chondrocytes were transfected with si-circFADS2 or si-NC and then treated with LPS. qPCR analysis was used to measure the miR-498 levels in each group. (**E**) Quantification of miR-498 and GAPDH mRNA by northern blot analysis. *P < 0.05, **P < 0.01 vs. control cells; ^#^ P < 0.05 vs. LPS group.

A previous study indicated that miR-498 is targeted by circFADS2 during lung cancer [18]. Thus, we hypothesized that circFADS2 could also target miR-498 and modulate its downstream functions in LPS-treated chondrocytes. Our hypothesis was supported by bioinformatic analyses (Figure 5A). The direct relationship between miR-498 and circFADS2 was investigated using the DLRA (Figure 5B). The function of luciferase was inhibited by 70 % in cells transfected with miR-498 mimic fused to the WT circFADS2 when compared to that of cells from the control groups. We further evaluated miR-498 expression in samples from RA patients and healthy controls. As shown in Figure 5C, the RA group expressed significantly less miR-498 than the healthy group. Additionally, miR-498 levels in LPS-treated chondrocytes were reduced when compared to those in non-treated cells using qPCR and Northern blotting detection (Figure 5D, 5E). Cells transfected with si-circFADS2 had significantly higher miR-498 levels.

### MiR-498 inhibitors restored the function of circFADS2 on LPS-induced chondrocytes.

**Figure 6:**
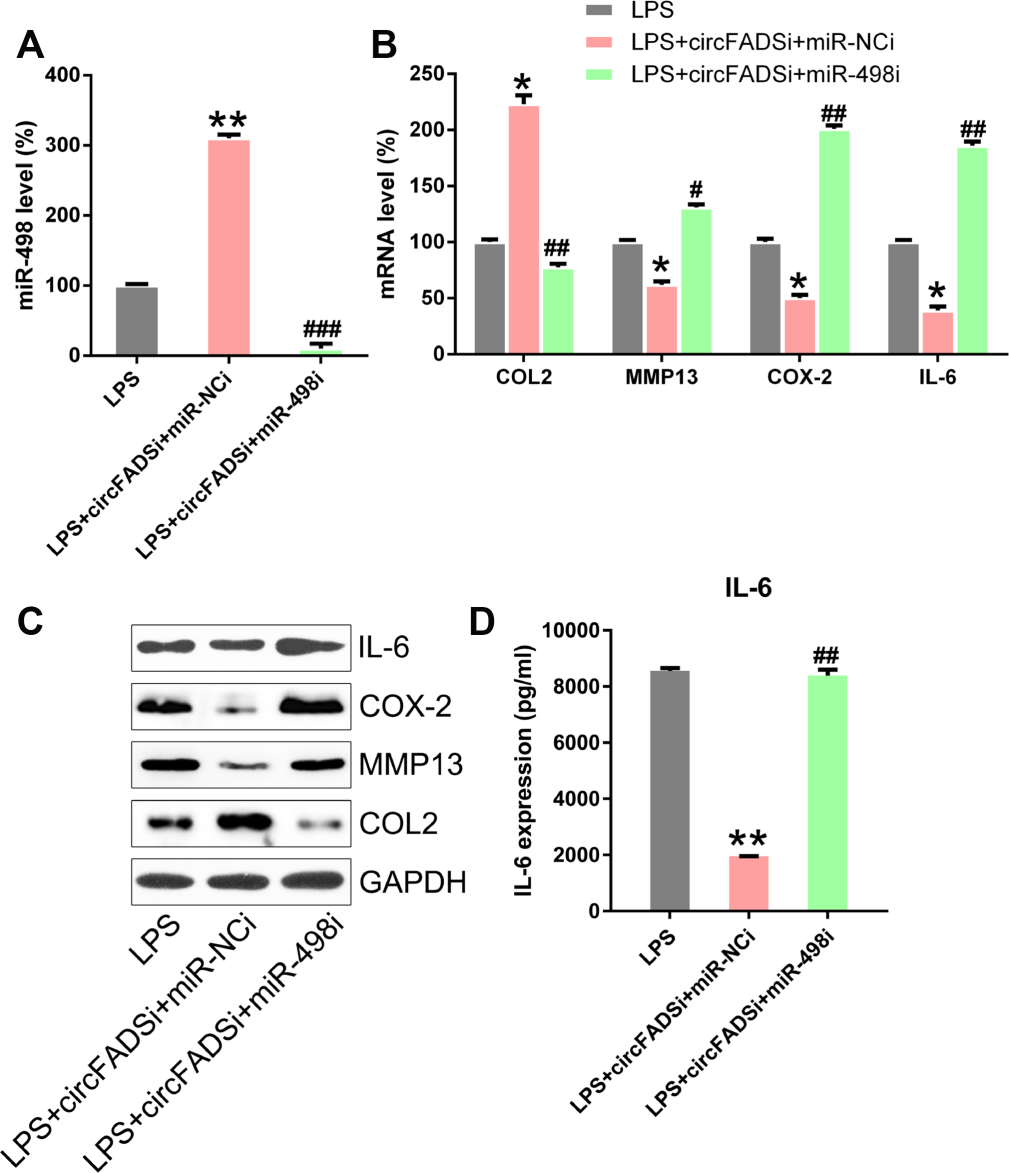
**miR-498 inhibitor reversed the effects of circFADS2 on LPS-treated chondrocytes.** LPS-treated chondrocytes were co-transfected with si-circFADS2 and miR-498 inhibitor/miR-NC inhibitor. (**A**) qPCR analysis was performed to confirm the miR-498 levels in each group. (**B**, **C**) The mRNA and protein expression of the indicated proteins was determined by qPCR and WB. (**D**) The IL-6 level in cells was examined by ELISA. Results were normalized to GAPDH expression. *P < 0.05, **P < 0.01 vs. LPS group; ^#^ P < 0.05, ^##^ P < 0.01, ^###^ P < 0.001 vs. LPS+circFADSi+miR-NCi group.

Furthermore, we explored whether miR-498 inhibitors could abolish the effects caused by the circFADS2 knock-down on LPS-induced chondrocytes or during RA progression. LPS-treated chondrocytes were co-transfected with si-circFADS2 and/or miR-498 inhibitors. The qPCR results confirmed that the levels of miR-498 were significantly downregulated in the circFADS2i+miR-498i group, compared those in the circFADS2i group (Figure 6A). WB, ELISA, and qPCR analyses demonstrated that silencing miR-498 restored the effect of circFADS2 on COL2, MMP13, COX-2, and IL-6 in LPS-induced chondrocytes (Figure 6B–6D).

**Figure 7:**
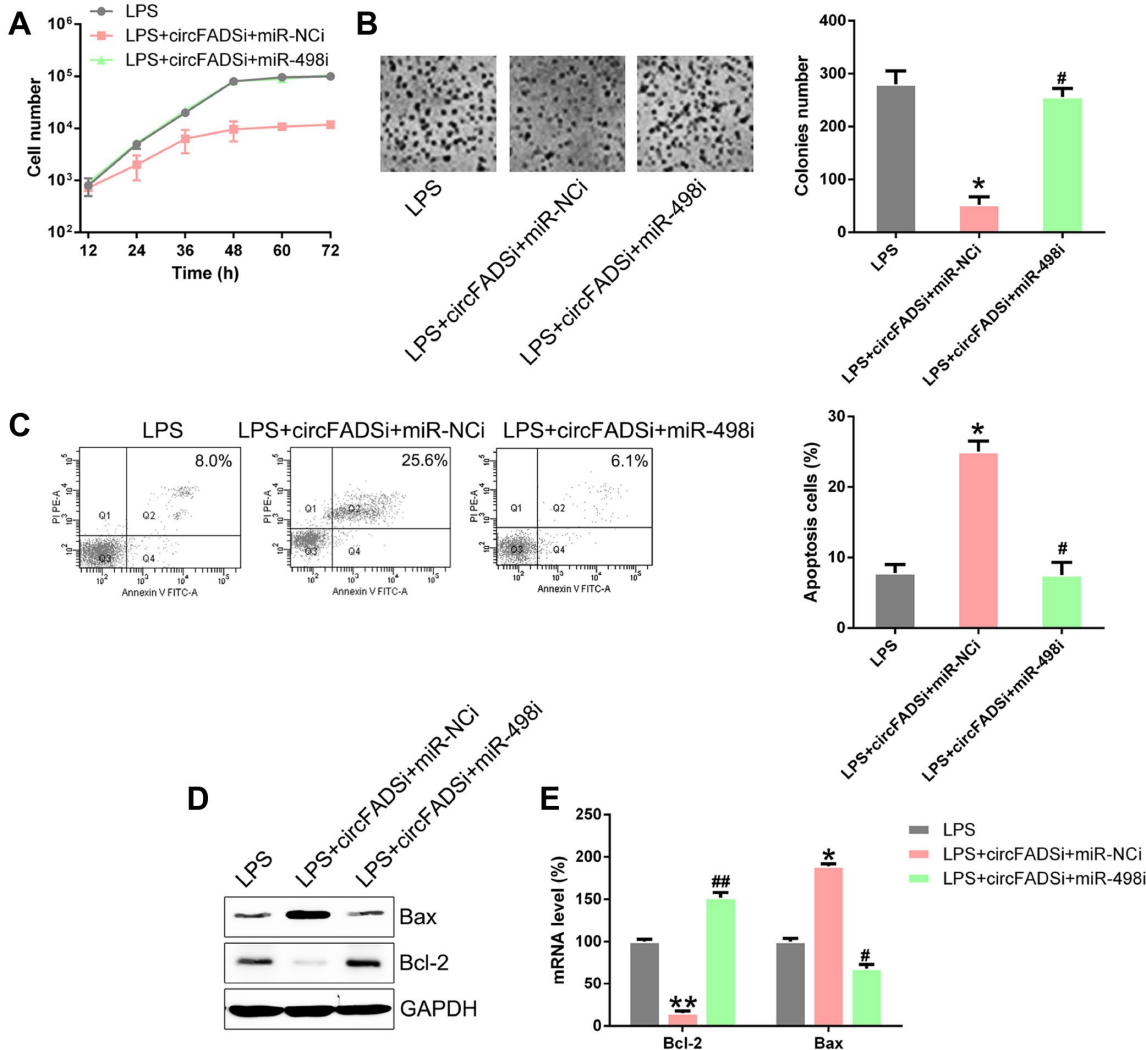
**miR-498 silencing reversed cell viability and apoptosis regulated by circFADS2.** LPS-treated chondrocytes were co-transfected with si-circFADS2 and miR-498 inhibitor/miR-NC inhibitor. (**A**) CCK8 assay showing that transfection with a miR-498 inhibitor reduced cell proliferation of LPS-treated chondrocytes. (**B**) Soft agar colony formation assay for the LPS-treated chondrocytes co-transfected with si-circFADS2 and miR-498 inhibitor/miR-NC inhibitor, and that of non-transfected LPS-induced cells. The right panel shows the number of colonies formed in each group. (**C**) Flow cytometry analysis showing the apoptosis levels in the different chondrocyte groups. (**D**) WB and (**E**) qPCR analysis showing that miR-498 inhibition upregulated Bcl-2 and decreased the levels of Bax, in LPS-treated chondrocytes. *P < 0.05, vs. LPS group; ^#^ P < 0.05, ^##^ P < 0.01 vs. LPS+circFADSi+miR-NCi group.

To evaluate the effect of miR-498 inhibition on cell proliferation and apoptosis, CCK-8 and colony generation assays were performed on the circFADS2i+miR-498i group, showing that miR-498 inhibitors caused a reduction in cell proliferation, similar to si-circFADS2 (Figure 7A, 7B). Moreover, flow cytometry showed that miR-498 inhibitors decreased the stimulatory effects of circFADS2i on apoptosis (Figure 7C). Additionally, WB analysis confirmed that transfection with miR-498 inhibitors reversed the expression levels of circFADS2i-regulated apoptotic factors (Bcl-2 and Bax) (Figure 7D, 7E). These data suggested that circFADS2 influences the properties of LPS-induced chondrocytes by regulating miR-498.

### MiR-498 regulates the properties of LPS-induced chondrocytes by targeting the mTOR gene

**Figure 8:**
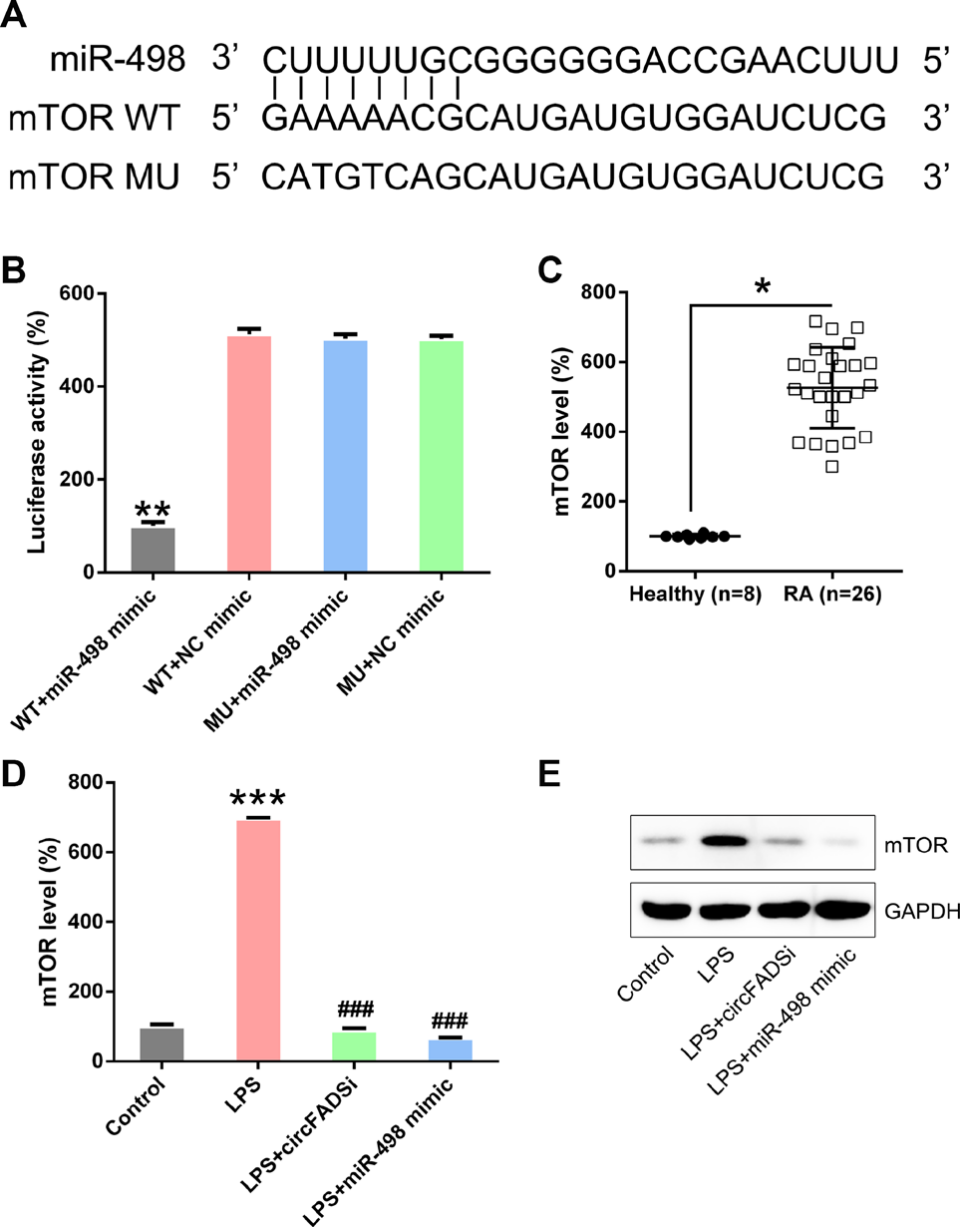
**miR-498 targets the mTOR.** (**A**) Bioinformatic analysis showing that miR-498 has a binding site in the 3′-UTR of mTOR. (**B**) DLRA was performed following co-transfection of chondrocytes with a luciferase reporter containing either a WT (wild-type) or MU (mutant) mTOR and a miR-498 mimic. (**C**) qPCR analysis showing the significantly increased mTOR expression levels in RA patients. (**D**, **E**) Chondrocytes were transfected with si-circFADS2 or miR-498 mimic and then treated with LPS. qPCR and WB analysis were carried out to detect mTOR levels in each group. *P < 0.05, **P < 0.01, ***P < 0.001 vs. control cells; ^#^P < 0.05, ^###^P < 0.001 vs. LPS group.

A predictive analysis revealed a possible binding site of miR-498 on the 3′-UTR of mTOR (Figure 8A), while DLRA showed that miR-498 mimic transfection reverses the decrease in luciferase activity through WT mTOR (Figure 8B), suggesting that miR-498 may target mTOR. mTOR was also remarkably up-regulated in RA patients when compared to the healthy group (Figure 8C). To further confirm the relationship between circFADS2, miR-498, and mTOR expression, circFADS2 was silenced, or miR-498 expression was upregulated in LPS-induced chondrocytes. We found that both circFADS2 silencing and miR-498 upregulation caused a significant reduction of mTOR expression at the protein and mRNA levels (Figure 8D, 8E).

**Figure 9:**
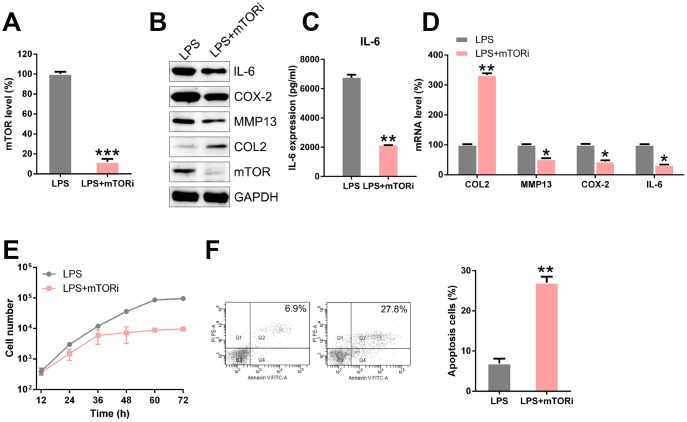
**mTOR inhibition regulates ECM degradation, inflammation, and apoptosis of LPS-treated chondrocytes*.*** LPS-treated chondrocytes were transfected with si-mTOR. (**A**) qPCR analysis was performed to confirm the mTOR mRNA levels in each group. (**B**) The protein levels of type II collagen, MMP13, COX-2, IL-6, and mTOR were determined by WB. (**C**) The IL-6 level in cells was examined by ELISA. (**D**) The mRNA levels of type II collagen, MMP13, COX-2, IL-6, and mTOR were determined by WB; results are normalized to the expression of GAPDH. (**E**) CCK8 assay showing that mTOR silencing inhibited cell proliferation of LPS-treated chondrocytes. (**F**) Flow cytometry analysis showing apoptosis levels in LPS-treated chondrocytes with mTOR silenced. *P < 0.05, **P < 0.01, ***P < 0.001 vs. LPS group.

To elucidate the role of mTOR in the processes of LPS-induced chondrocytes, such as ECM disorder, inflammation, and apoptosis, we performed the experiment mentioned above in LPS-induced chondrocytes with a mTOR knockdown. qPCR and WB analysis confirmed that mTOR was downregulated in the transfected cells (Figure 9A, 9B). The results showed that mTOR silencing increased the expression of COL-2 and reduced the levels of MMP13, COX-2, and IL-6 (Figure 9B–9D). Additionally, mTOR inhibition decreased the proliferation of LPS-induced chondrocytes (Figure 9E) and increased the apoptotic levels in LPS-treated chondrocytes (Figure 9F). These data suggested that circFADS2 and miR-498 may exert their functions via mTOR.

## DISCUSSION

LPS is the main component of the cell wall of G-bacteria, which could induce severe inflammation response [19, 20], so we used LPS to build a well-established model for in vitro inflammatory investigation. Meanwhile, there were accumulating studies using LPS to establish an artificial “RA cell model”, but this LPS-induced “RA model” is still an artificial RA model [21, 22]. In this study, we explored the role of circFADS2 in RA using LPS-treated chondrocytes artificial model since previous studies have revealed that LPS emergence may represent the initial stage of inflammation in RA pathogenesis. [20]. Through this study, we found that, after LPS treatment, chondrocytes collected from the cartilage of rats up-regulated circFADS2, and inferred that circFADS2 might play an essential role in regulating LPS induced inflammation in chondrocytes.

Chondrocytes are critical in balancing the anabolism and catabolism of ECM, and RA is frequently worsened by the imbalance induced by ECM degradation [23, 24]. Additionally, anomalies in COL2 production and MMP13 expression also contribute to OA. This disease triggers severe inflammation mediated by an increase in COX-2 and IL-6 [25, 26]. To further evaluate the role of circFADS2, we transfected rat-isolated chondrocytes with si-circFADS2 after LPS treatment. Our results showed that circFADS2 silencing reversed the increase in MMP13 levels induced by LPS treatment and caused an increase in COL2 levels. Besides, after circFADS2 was silenced, COX-2 and IL-6 were significantly down-regulated in comparison to the LPS-control chondrocytes.

MiR-498 has been implicated in several types of cancer, like ovarian cancer and esophageal squamous cell cancer [27, 28]. It was found that miR-498 expression was reduced and that STAT3 was increased in PMBCs of RA patients. miR-498 overexpression inhibits the STAT3-mediated differentiation of PBMC Th17 cells in RA patients [29]. However, the mechanisms of miR-498 involvement in RA are not fully understood. Thus, we needed to determine whether miR-498 related to circFADS2 in RA progression. Our results indicated that miR-498 is a direct target of circFADS2 and that circFADS2 levels are negatively related to miR-498 levels in LPS-treated chondrocytes. Moreover, a decrease in miR-498 abolished the protection caused by circFADS2 in ECM catabolism, inflammation, and apoptosis of chondrocytes. In conclusion, the protective effect of circFADS2 on LPS-treated chondrocytes is mediated, at least partially, by miR-498.

The PI3K/AKT/mTOR axis has been shown to be critical in RA pathogenesis and symptom mitigation of RA patients [30]. As an intracellular signaling pathway, it is involved in a variety of biological events, including apoptosis [31, 32] and inflammation [33, 34]. A previous report indicated that artesunate increased cell apoptosis in rat models with RA by suppressing the activity of the PI3K/AKT/mTOR axis [35]. In the present study, after silencing mTOR in LPS-treated chondrocytes, we found that mTOR played a similar role in ECM catabolism, inflammation, and apoptosis than circFADS2, which was also in contrast to the function of miR-498.

Bioinformatic analyses predicted a putative region of interaction of circFADS2-miR-498-mTOR, which was confirmed by DLRA. qPCR revealed that the down-regulation of miR-498 was negatively related to circFADS2 and mTOR expression in LPS-treated chondrocytes. CircFADS2 silencing significantly increased miR-498 expression, while miR-498 mimic transfection caused a decrease in mTOR expression in LPS-treated chondrocytes. In conclusion, our data suggested that circFADS2 protects LPS-treated chondrocytes from apoptosis and inflammation acting as an interceptor of the miR-498/mTOR cross-talking.

## METHODS

### Cell origin and culture

Specific-pathogen-free (SPF), Sprague Dawley (SD) male rats weighing between 200 and 250 g were purchased from Clinical Medical College, Yangzhou University. Chondrocytes were isolated as previously described [21]. After isolation of cartilage, the tissue was subjected to three washes with DMEM and sliced into pieces of 1 mm^3^, followed by collagenase A digestion overnight at 37 °C. When cells reached 80% confluence for the first time, cells were divided into two dishes and further cultured until they reached confluence for the second time. Lipopolysaccharide (LPS) treatment was performed by adding 10 μg/mL LPS to the cells.

### Subjects

Subjects were selected from the patients that were admitted to Clinical Medical College, Yangzhou University, for the first time. Eligible subjects conformed to the diagnostic criteria of RA, did not show any complications, and had not previously been treated with immunosuppressants. We also recruited 8 healthy volunteers to serve as the control group (male = 4, female = 4). We collected the samples of cartilage tissues from 26 RA patients and 8 healthy volunteers.

Prior to the study, the protocols had been reviewed and approved by the Ethics Committee of Clinical Medical College, Yangzhou University, and all patients and their families signed informed consents.

### Total RNA isolation

Total RNA was isolated from cartilage tissues using a TRIzol kit (Invitrogen, Carlsbad, CA, USA) in accordance with the manufacturer’s instructions. Total RNA was isolated using an RNAprep Pure FFPE Kit in accordance with the manufacturer’s instructions (TIANGEN, Beijing, China). The purity and concentration of the total RNA samples were measured with a NanoDrop ND-1000 (Thermo Fisher Scientific, Wilmington, DE, USA) by absorbance measurements at 260 nm, 280 nm and 230 nm. Specifically, OD260/OD280 ratios between 1.8 and 2.1 were deemed acceptable, while OD260/OD230 ratios of greater than 1.8 were deemed acceptable. RNA integrity and contamination were tested by denaturing agarose gel electrophoresis. RNA was prepared and stored at −80 °C for the validation experiments.

### Microarray and quantitative assessment

Isolated cartilage tissues from 3 RA patients and 3 healthy volunteers were shock-frozen at once with the help of liquid nitrogen. The specimens were homogenized with TRIzol reagent (Invitrogen). NanoDrop ND-1000 was used to quantify total RNA in every specimen. Specimens were prepared and microarray hybridization was carried out according to Arraystar standard protocols. In short, total RNA in every specimen was amplified and transcribed to fuorescent cRNA using random primers, as per Arraystar Super RNA Labeling protocol (Arraystar Inc.). Arraystar Human circRNA Array (8× 15 K, Arraystar) was used to hybridized the labeled cRNAs. Agilent G2505C Scanner was used to scan arrays afterwashing the slides. Array images were evaluated using Agilent Feature Extraction software (version 11.0.1.1). R software package was used to normalize data and for analyses.

### qPCR detection

To detect the expression profiles of circRNA, circRNAs (hsa_circ_0022387, circFADS2, sequence: 5′-GGG TGC CTC TGC CAA CTG GTG GAA TCA TCG CCA CTT ATT CGT GGG CAA GTG CTT GAA ACC CAG CAC CAC GCC AAG CCT AAC ATC TTC CAC AAG GAT CCC GAT GTG AAC ATG CTG CAC GTG TTT GTT-3′) were selected for qPCR assay as follows. After RNA extraction from the samples, cDNA was synthesized with a reverse transcriptase according to the kit’s instructions (SuperScript First-Strand Synthesis System for RT-PCR, Invitrogen, Carlsbad, CA, USA). Divergent primers of hsa_circ_0022387 was designed with Primer Premier software version 5.0 (Premier Biosoft International, Palo Alto, CA, USA), ensuring that circRNAs were amplified through head-to-tail splicing. GAPDH, a housekeeping gene, was used as internal standard for normalization. Primers were synthesized by Bioligo Biotech (Shanghai, China). The sequences of hsa_circ_0022387 and GAPDH primers were as follows: 5′-GCC AAC TGG TGG AAT-3′ (forward) and 5′-GTG CAG CAT GTT CAC-3′ (reverse) for hsa_circ_0022387; and 5′-GTG GCC GAG GAC TTT G-3′ (forward) and 5′-CCT GTA ACA ACG CAT CT-3′ (reverse) for GAPDH. PCR was conducted in a 10-μl reaction volume consisting of the following: 2.0 μl cDNA, 5 μl 2 × PCR Master mix (PCR Master Mix, Arraystar, Rockville, MD, USA), 0.5 μl primer forward (5 μM), 0.5 μl primer reverse (5 μM), and 2.0 μl H2O. The qPCR reaction was performed on an ABI QuantStudioTM 5 System (Applied Biosystems, DE, USA) as follows: initial denaturation at 95 °C for 10 min, 40 cycles of amplification at 95 °C for 10 sec, annealing and extension at 60 °C for 1 min. Amplification products were analyzed by 1.5% agarose gel electrophoresis, stained with 1:10000 dilution of GelRed Nucleic Acid gel stain (Biotium, CA, USA) and visualized under ultraviolet illumination for band size consistency. All the experiments were conducted in triplicate. The data were analyzed by using the comparative cycle threshold (2^−ΔΔCt^) to represent a relative expression level of circRNAs.

### Northern blotting

5 to 10 μg total RNA was fractionated on 1.5% formaldehyde agarose gels and transferred to Zetaprobe membrane (Biorad Inc, Hercules, CA, USA). Membranes were washed overnight at 55°C with SSC (0.3M NaCl and 0.03M Na citrate [pH 7.0]) and 1% sodium dodecyl sulfate and prehybridized for a minimum of 4 hrs with ULTRAhyb oligonucleotide hybridization buffer (Ambion). The oligonucleotides of probes used here were same as qPCR assay. The probes (30 pmol) were labeled with [γ-^32^P] ATP using T4 polynucleotide kinase (New England Biolabs, MA, USA). Membranes were hybridized overnight at 37°C in ULTRAhyb oligonucleotide hybridization buffer and washed the following morning 3 times with SSC at 37°C. Washed membranes were subjected to phosphorimage analysis (Molecular Dynamics, CA, USA).

### Western blotting

Tissues were placed in lysis buffer for protein extraction. Following the measurement of protein concentration, protein samples (the same amount in each sample) were loaded onto SDS-PAGE and separated by electrophoresis. The proteins on the gels were electrically transferred to nitrocellulose (NC) membranes (Roche Biosciences, Germany) for 2 h at 4 °C. The NC membrane was then blocked, incubated in the presence of primary antibodies at 4°C overnight, and then probed using secondary antibodies at room temperature for 1 h. Bands on the membrane were developed using the ECL reagent (ECL, Pierce).

### Enzyme-linked immunosorbent assay (ELISA)

The tested proteins in cells were measured with Human IL-6 immunoassay (R&D systems, Minneapolis, MN), following the manufacturer’s instructions, respectively.

### CircRNA and miRNA plasmid construction and transfection

The small interfering RNAs (si-circFADS2, 5′-CAC GAA TAA GTG GCG ATG ATT-3′ and si-NC, 5′-GAT GAC TAA CAT ATG GTC ATT-3′) were purchased from GenePharma (Shanghai, China). MiR-498 mimics, miR-498 inhibitors, and the corresponding mimic/inhibitor negative control (NC) oligos were provided by Ribobio (Guangzhou, China). The chondrocytes isolated from the rats were transfected with siRNAs or miR-498 inhibitors/mimics using Lipofectamine 2000 (Invitrogen).

### Cell counting Kit-8 (CCK-8) assay

Cell counting kit-8 (CCK-8, Dojindo) was used to determine cell proliferation. The cells were cultured for 3 days in a 96-well plate and treated with 10 μL of CCK-8 solution for 2 h. The absorbance of the sample was measured at a wavelength of 450 nm. Proliferation rates were determined at 12-72 h after transfection.

### Colony generation assay

The cell suspension was prepared using DMEM supplemented with 10% FBS 2 days after transfection and plated on an 8-mm layer of 0.4% top agar; after 24 hrs, cells were transferred to 12-well plates. After 14 days, four regions were randomly chosen from each plate and colonies were quantified.

### Analysis of apoptosis

The characterization of apoptotic cells was performed using Annexin V/propidium iodide (PI) staining and flow cytometry (FC) assay as previously described [36]. Briefly, a total of 1×10^5^ cells from each group were collected by centrifugation (2000 rpm, 5 min) and washed with PBS. Cells were resuspended in PBS with 1% bovine serum albumin and 1% FBS, followed by 20 min incubation in the dark with PI (5 μL) and annexin V-FITC (10 μL) at room temperature in the dark. Assay results were measured using the Guava flow cytometry system. Results were expressed as percentage of apoptotic cells, and error bars represented SD value.

### Dual-luciferase reporter assay (DLRA)

For the luciferase reporter assay, the pGL3 plasmid encoding a luciferase reporter gene was purchased from Promega Corporation (Madison). Recombinant plasmid of pGL3-circFADS2-wild-type (WT) and mutant (MU), as well as pGL3-mTOR-WT and MU were constructed. HEK-293T cells were co-transfected with miR-498 mimics or miR-NC, pGL3-circFADS2/mTOR-WT and MU using Lipofectamine 2000 (Invitrogen). The pRL-TK vector was used as a normalization control. After transfection for 48 h at 37°C, cells were harvested and assayed with a Dual-Luciferase Reporter Assay system (Promega) according to the manufacturer’s protocol. The reporter luminescence (Rluc) was studied using the sequence of Renilla luciferase, while the calibration luminescence (Luc) was assayed using the sequence of the firefly luciferase.

### TargetScan prediction

We used the prediction algorithm TargetScan to nominate targets of miR-498. Using the TargetScan Database predictions (http://www.targetscan.org) are ranked based on the predicted efficacy of targeting as calculated using cumulative weighted context++ scores of the sites [37]. As an option, predictions are also ranked by their probability of conserved targeting [38].

### Statistical analysis

The data obtained in this study are expressed as means ± standard deviation (SD). Comparisons between the two test groups were performed using the one-way analysis of variance (ANOVA), along with the two-tailed Student’s t-test. *P* < 0.05 indicated statistically significant differences between the groups.
